# Combined effects of 17β-estradiol and exercise training on cardiac apoptosis in ovariectomized rats

**DOI:** 10.1371/journal.pone.0208633

**Published:** 2018-12-20

**Authors:** Yi-Yuan Lin, Jwo-Sheng Chen, Xu-Bo Wu, Woei-Cherng Shyu, Rungchai Chaunchaiyakul, Xian-Li Zhao, Chia-Hua Kuo, Yu-Jung Cheng, Ai-Lun Yang, Shin-Da Lee

**Affiliations:** 1 Departmental of Rehabilitation, Seventh People’s Hospital Affiliated to Shanghai University of Traditional Chinese Medicine, Shanghai, China; 2 Graduate Institute of Clinical Medical Science, China Medical University, Taichung, Taiwan; 3 Department of Sports Medicine, China Medical University, Taichung, Taiwan; 4 School of Rehabilitation Science, Shanghai University of Traditional Chinese Medicine, Shanghai, China; 5 Graduate Institute of Biomedical Sciences, China Medical University, Taichung, Taiwan; 6 Translational Medicine Research Center, China Medical University Hospital, Taichung, Taiwan; 7 College of Sports Science and Technology, Mahidol University, Salaya, Thailand; 8 Department of Sports Sciences, University of Taipei, Taipei, Taiwan; 9 Department of Physical Therapy, Graduate Institute of Rehabilitation Science, China Medical University, Taichung, Taichung, Taiwan; 10 Department of Occupational Therapy, Asia University, Taichung, Taiwan; Institute of Biochemistry and Biotechnology, TAIWAN

## Abstract

**Background:**

The purpose of this study was to investigate the combined 17β-estradiol (E2) and exercise training on cardiac pro-survival and anti-apoptotic pathways in ovariectomized rats.

**Methods:**

Fifty-six female Sprague–Dawley rats were divided into a sham-operated (Sham), a bilaterally ovariectomized (OVX), an OVX treated with E2 (OVX-E2; 10μg/kg/day), and an OVX with E2 and treadmill exercise training (OVX-E2-EX; 60 min/day, 5 days/week) for 10 weeks. Following 10 weeks of exercise training, rat hearts were isolated for the evaluation of Histopathological analysis, TUNEL assay, and Western blotting.

**Results:**

The protein levels of estrogen receptor α (ERα), estrogen receptor β (ERβ), insulin-like growth factor 1 (IGF-1), IGF-1 receptor (IGF-1R), phospho-phosphatidylinositol 3-kinase (p-PI3K) (estrogen receptors/IGF-1-related survival pathway) were significantly increased in either the OVX-E2 or OVX-E2-EX group when compared with the OVX group. The protein levels of B-cell lymphoma 2 (Bcl-2), B-cell lymphoma-extra-large (Bcl-xL) and phosphorylated-Bad (p-Bad) (Bcl-2 family survival pathway) were significantly increased in the OVX-E2-EX group when compared with the OVX group. Only the p-Bad was significantly increased in the OVX-E2 group when compared with the OVX group. The protein levels of truncation of Bid (t-Bid), Bcl-2-associated death promotor (Bad), Bcl-2-associated X protein (Bax), Cytochrome *c*, caspases-9, and caspases-3 (mitochondria-dependent apoptotic pathway), as well as the protein levels of tumor necrosis factor-α (TNF-α), Fas ligand, Fas receptors, Fas-associated death domain (FADD), activated caspase-8 and activated caspase-3 (Fas receptor–dependent apoptotic pathway) were significantly decreased in either the OVX-E2 or OVX-E2-EX group when compared with the OVX group. Furthermore, when compared with the OVX-E2 group, the protein levels of ERβ, IGF-1, IGF-1R, Bcl-2 and Bcl-xL were further enhanced in the OVX-E2-EX group as well as the protein levels of Cytochrome *c*, Fas receptors, FADD, activated caspase-8, activated caspase-9 and activated caspase-3 were further decreased in the OVX-EX-E2 group.

**Conclusions:**

Combined E2 and exercise training exhibited a positive effect of protection on ovariectomy-induced cardiac apoptosis by enhancing ERβ-related survival pathways, which might provide a more effective therapeutic effect on cardiac protection in bilaterally oophorectomized or menopausal women than E2 treatment only.

## Introduction

According to findings which indicated that women who enter menopause early may be have a higher risk for cardiovascular disease and premature death [1]. Estrogen deficiency at menopause could cause left ventricular hypertrophy and systolic dysfunction, which have been associated with increased cardiac apoptosis, as well as potentially develop heart failure [2, 3].

The cardiomyocyte apoptosis is one of very critical pathological mechanisms to cause heart failure, which can be regarded as marker of poor cardiovascular outcomes [4, 5]. The apoptotic cascade is triggered mitochondria-dependent (intrinsic) and Fas receptor-dependent (extrinsic) apoptotic pathways [6]. The mitochondria-dependent apoptotic pathway is initiated from truncation of Bid (t-Bid) induce the oligomerization of Bcl-2-associated X protein (Bax) and Bcl-2-associated death promotor (Bad). These pro-apoptotic proteins (t-Bid, Bax, and Bad) can enhance release of Cytochrome *c* into the cytosol, which is responsible for the activation of caspases-9 and caspases-3 [6, 7]. Pathways initiated from the Fas ligand and tumor necrosis factor alpha (TNF-α) binding stimulates trapping of the Fas-receptors, starting with the recruitment of a death-inducing signaling complex (DISC) via Fas-associated death domain (FADD), which is responsible for the activation of caspases-8 and leads to caspase-3 cleavage and executes the cell death program [6]. Our previous studies showed that cardiac Fas-dependent and mitochondria-dependent apoptosis were acticated after ovariectomy [8].

Estrogen not only acts through estrogen receptors (ERα and ERβ), but can also bind to membrane-bound receptors that can cause some of the effects reported [9]. Estrogen is able to mediate stimulation of the IGF-1 receptor (IGF-1R) pathway and phosphorylate IGF-1 receptor (pIGF-1R) [10]. IGF-1 signaling contributes to regulating cardiomyocyte survival through PI3K and Akt activity, which indirectly enhances the Bcl-2 anti-apoptotic family B-cell lymphoma 2 (Bcl-2), B-cell lymphoma-extra-large (Bcl-xL) and phosphorylated-Bad (p-Bad), and prevents apoptosis [11]. Furthermore, 17β-estradiol (E2) has been shown to suppress cardiac apoptosis through activation of the phosphatidylinositol 3-kinase (PI3K)/protein kinase B (Akt) pathway during myocardial infarction [12]

E2 is the most abundant ovarian estrogen and may provide cardiovascular protection [13]. A previous study showed that E2 has an anti-apoptotic effect in rat models of myocardial infarction [12]. Moreover, E2 treatment has been shown to attenuate cardiac apoptosis, cardiac remodeling and reducing interstitial myocardial fibrosis after an ovariectomy or aging-related defects [14, 15]. Nevertheless, several research suggested that long-term E2 therapy is associated with an increased risk of breast cancer, or severe menopausal side effects in some women [16, 17].

Physical exercise is a well-known approach for the prevention and treatment of cardiovascular diseases in cardiac rehabilitation [18, 19], and aerobic exercise training has benefits for cardiovascular adaptation in postmenopausal women [20–22]. Our previous study reported that exercise training can significantly prevent cardiac apoptosis in ovariectomized rats [23]. Furthermore, physical activity can also lower the risk of developing breast cancer [24]. However, the combined effect of E2 treatment and exercise training on cardiac apoptosis in early oophorectomized or postmenopausal women is unclear. We hypothesize that the combined effects of E2 treatment and exercise training might have positive effect and more effective than E2 treatment only in preventing mitochondria-dependent and Fas receptor-dependent apoptotic pathways.

## Materials and methods

### Animals

Fifty-six female Sprague–Dawley rats were used in this study. The rats were given free access to standard chow (Lab Diet 5001; PMI Nutrition International, Brentwood, MO), and water *ad libitum*. This study protocol designs and animal care were performed in compliance with the ethics guideline for the care and use of Laboratory animals followed by the National Institutes of Health. All procedures were approved by the Institutional Animal Care and Use Committee of the China Medical University and the Cheng Kung University Animal Center (Ethical approval code: 95121).

### Ovariectomy and sham operation

Bilateral ovariectomy and sham operations were performed according to the technique described by Huang [23]. At fourteen-week-old, rats were randomly assigned to the sham-operated rats (Sham, n = 14) and bilateral ovariectomized rats (n = 42). All rats underwent survival surgical procedures and anesthetized with a mixture of ketamine and xylazine (100:10 mg/kg body weight; i.p.). The sham-operated rats underwent the same surgical procedure, but the bilateral ovaries were not removed. The bilateral ovaries were removed in the ovariectomized rats. After surgery, each rat was injected with 0.2cc Penicillin-G procaine (20,000 IU; IM) and allowed recovery times of at least one week. The ovariectomized rats were further divided into an untreated sedentary group (OVX, n = 14), a group treated with 17β-estradiol (OVX-E2, n = 14) and a group treated with 17β-estradiol and exercise (OVX-E2-EX, n = 14).

### Administration of 17β-estradiol

The protocols for administered 17β-estradiol were conducted according to previous study [14]. The OVX-E2 and OVX-E2-EX groups were injected intraperitoneally with 10 μg/kg body weight/day of 17β-estradiol (E2; Sigma Chemical Company, St. Louis, MO, USA) dissolved in sesame oil (Sigma Chemical Company) for 10 weeks. The Sham group was injected intraperitoneally with equal volume sesame oil (about 0.3cc) as the vehicle for 10 weeks.

### Exercise training

The OVX-E2-EX group were adapted to the treadmill training protocol on a motor-driven leveled treadmill (Treadmill Exerciser T408E, Diagnostic and Research Instruments Co., Taoyuan, Taiwan) were performed according to our previous study [23]. At first day, the rats were familiarized with treadmill speed at a speed of 12 m/min for 20 min. Subsequently, treadmill initial speed of 15m/min, gradually increased by 3 m/min every two weeks until treadmill speed of 24 m/min for 60 min/day, 5 days/week, for 10 weeks. The sedentary Sham and OVX groups were placed on the treadmill running apparatus without exercise training for the same environmental stimulation. At the end of the experimental period, twenty-five-week-old rats were sacrificed and anaesthetized with 2% inhaled isoflurane delivered in oxygen (5% CO_2_ and 95% O_2_).

### Cardiac characteristics

The hearts of rats in the four groups were excised and cleaned in phosphate-buffered saline (PBS, pH 7.4). The left ventricles were separated from whole hearts. The right tibia length (TL), the whole heart weight (WHW) and the left ventricle weight (LVW) were measured. The ratio of left ventricle weight to whole heart weight, whole heart weight to body weight, left ventricle weight to body weight, whole heart weight to tibia length and left ventricle weight to tibia length were calculated.

### Hematoxylin-Eosin staining

The hearts of six rats per group were soaked in 4% paraformaldehyde. Paraffin-embedded heart sections were cut into 2-μm thick. Hematoxylin-eosin staining was performed as previously reported [25]. In brief, the heart sections were de-paraffinized in xylene and rehydrated through graded alcohols (100%, 90%, 85% and 75%) and then rinsed in PBS (pH 7.2). All heart sections were stained with hematoxylin and eosin (Merck, Darmstadt, Germany), then dehydrated in graded alcohols (75%, 85%, 90% and 100%), and followed by placed in xylene. Photomicrographs in Hematoxylin-Eosin staining, Masson’s trichrome staining, and TUNEL assay were captured under a phase contrast microscope connecting to a digital camera (Olympus BX43, Tokyo, Japan).

### Masson’s trichrome staining

The heart sections were also stained with Masson’s trichrome stain kit (ScyTek Laboratories, Inc., UT, USA) as previously described [25]. Brief, the heart sections were incubated with Bouin solution at 60°C for 45 min, washed in running tap water until the yellow color disappeared. Then each slide was immersed in Weigert’s Haematoxylin solution for 5 min, washed in running tap water. After that, each slide was stained for 15 min with Acid Fuchsin, rinsed in distilled water. The slides were incubated for 10 min with a phosphomolybdic acid solution and then immediately stained for 10 min with methyl blue solution, rinsed in distilled water. Then slides were treated with 1% acetic acid solution for 3 min or until bluish collagen fibers were observed. Each slide was dehydrated through two changes of 95% alcohol followed by two changes of 100% alcohol, and then placed in xylene. The quantification of fibrotic areas (stained blue) and myocardial areas (stained red) was measured by ImageJ analysis software. The fibrosis percentage was calculated by ratio of fibrotic area to the myocardial area.

### DAPI and TUNEL staining

For terminal deoxynucleotidyl transferase dUTP-mediated nick-end labeling (TUNEL) assay using an In Situ Cell Death Detection Kit Fluorescein (Roche Applied Science) as previously described [25]. Briefly, the slides were treated with proteinase K for 30 min, washed in PBS, and then incubation with permeabilisation working solution (0.1% Triton X-100 in 0.1% Sodium Citrate, freshly prepared) for 8 min, washed in PBS. The slides were incubated with a blocking buffer for 60 min, washed in PBS. The slides were incubated with terminal deoxynucleotidyl transferase and fluorescein isothiocyanate-dUTP conjugated in the dark at 37 °C for 60 min and then washed in PBS. Next, the slides were mounted using DAPI-fluoromount G (Southern Biotech, Birmingham, AL, USA). TUNEL-positive nuclei (fragmented DNA) under a fluorescence microscope at an excitation wavelength in the range of 450–500 nm and a detection the green fluorescence in the range of 515–565 nm. The nucleus position under a fluorescence microscope at an excitation wavelength in the range of 340–380 nm and blue emission wavelength of 435-485nm. The numbers of DAPI-stained nuclei and TUNEL-positive were determined, and the percentage of TUNEL-apoptosis was calculated by ratio of (TUNEL-positive numbers) /(DAPI-stained nuclei numbers) ×100%.

### Tissue extraction

The left ventricular tissues of eight rats from each group were excised and cleaned with ice-cold saline, followed by homogenized in tissue protein extraction reagent (T-PER, Thermo Scientific, Rockford, IL, USA) containing phosphatase and/or protease inhibitors cocktail (100×, Roche Applied Science, Mannheim, Germany) at 4 °C. The supernatant was collected by sequentially centrifuged at 12,000g for 40 min.

### Western immunoblotting

Protein concentration of the heart tissue was determined by the Bradford method (Bio-Rad Laboratories, Hercules, CA, USA). Protein samples (40μg in each well) were separated on a 12% sodium dodecyl sulfate–polyacrylamide gel electrophoresis with a constant voltage of 75 V, and then transferred to polyvinylidene difluoride membrane (PVDF, 0.45-μm pore size, Millipore, Bedford, MA, USA) with a transfer apparatus (Bio-red, Hercules, CA, USA). PVDF membranes were incubated with blocking buffer containing 5% non- fat dried milk powder in TBST buffer (25 mM Tris-HCl, 150 mM NaCl, pH 7.6, and 0.1% Tween-20) followed by incubation with primary antibodies were diluted to 1:500 in antibody binding buffer overnight at 4°C. Primary antibodies including ERα, ERβ, IGF-1, IGF-1R, p-PI3K, p-Akt, Bcl-2, Bcl-xL, p-Bad, t-Bid, Bad, Bax, Cytochrome c, TNF-α, Fas ligand, Fas receptors, FADD, caspase-8, caspase-9, caspase-3 and α-tubulin (Santa Cruz Biotechnology, Santa Cruz, CA, USA). The membranes were washed in TBST buffer, and followed by incubation with a horse-radish peroxidase conjugated second antibody solution (1:5000 diluted in antibody binding buffer, Santa Cruz Biotechnology) for 60 min at room temperature, washed in TBST buffer. The immunoblotted proteins were visualized using the Immobilon Western Chemiluminescent HRP Substrate Reagent (Millipore Corporation, Billerica, MA, USA) and blot intensity was quantified using a chemiluminescence detection system (Fujifilm LAS-3000, Tokyo, Japan).

### Statistical analysis

All data of body weight, heart weight indices, TUNEL (+) apoptosis %, cardiac fibrosis % and protein levels were compared among the Sham, OVX, OVX-E2, and OVX-E2-EX groups using one-way ANOVA with pre-planned contrast comparison with the control group and then do Tukey’s HSD post hoc tests. The Sham and OVX served as the negative control and the positive control, respectively. *p* < 0.05 was considered to be significant.

## Results

### Body weight and cardiac characteristics

The OVX group weighed about 35% more than the age-matched Sham group, whereas those were significantly decreased in the OVX-E2 (8.4%) or OVX-E2-EX (20%) groups when compared with the OVX group (Table 1). The whole heart weight (WHW), left ventricular weight (LVW), the ratio of whole heart weight to tibia length (WHW/TL) and the ratio of left ventricular weight to tibia length (LVW/TL) in the OVX group were higher than those in the Sham group (Table 1), whereas the WHW, LVW, WHW/TL and LVW/TL in the OVX-E2 or OVX-E2-EX groups were lower than those in the OVX group, and even greater decreases were observed in the OVX-E2-EX group when compared with the OVX-E2 group (Table 1).

**Table 1:** Cardiac characteristics of sham, OVX, OVX-E2 and OVX-E2-EX groups.

	Sham	OVX	OVX-E2	OVE-E2-EX
***Number of animals***	8	8	8	8
***Body weight (g)***	287±13	390±9**	357±18**^##^	312±19*^##^^††^
***Heart Weight Index***				
WHW (g)	0.84±0.05	1.01±0.02**	0.95±0.04**^#^	0.86±0.02^##^^††^
LVW (g)	0.61±0.04	0.73±0.02**	0.69±0.03**^##^	0.64±0.02^##^^††^
LVW (g) / WHW (g)	0.73±0.10	0.73±0.03	0.73±0.06	0.74±0.03
WHW (g) / BW (kg)	2.95±0.24	2.59±0.09**	2.66±0.10**	2.77±0.20
LVW (g) / BW (kg)	2.12±0.18	1.89±0.06*	1.93±0.17*	2.05±0.11
WHW (g) / TL (mm)	23.61±1.44	27.28±0.63**	25.81±1.11**^#^	23.72±0.76^##^^††^
LVW (g) / TL (mm)	17.01±1.20	19.90±0.59**	18.69±0.85**^#^	17.58±0.39^##^^†^

Values are means ± SD. Four groups: sham-operated (Sham), bilateral ovariectomized (OVX), OVX treated with 17β-estradiol (OVX-E2) and OVX rats treated with 17β-estradiol and exercise (OVX-E2-EX). BW, body weight; WHW, whole heart weight; LVW, left ventricular weight; TL, Tibia length.

*P<0.05,

**P<0.01, indicate significant differences from the Sham group.

^#^*P*<0.05,

^##^*P*<0.01, indicate significant differences from the OVX group.

^†^*P*<0.05,

^††^*P*<0.01, indicate significant differences between the OVX-E2 and OVX-E2-EX group.

### Cardiac histopathological changes and TUNEL-positive apoptotic cells

**Fig 1:**
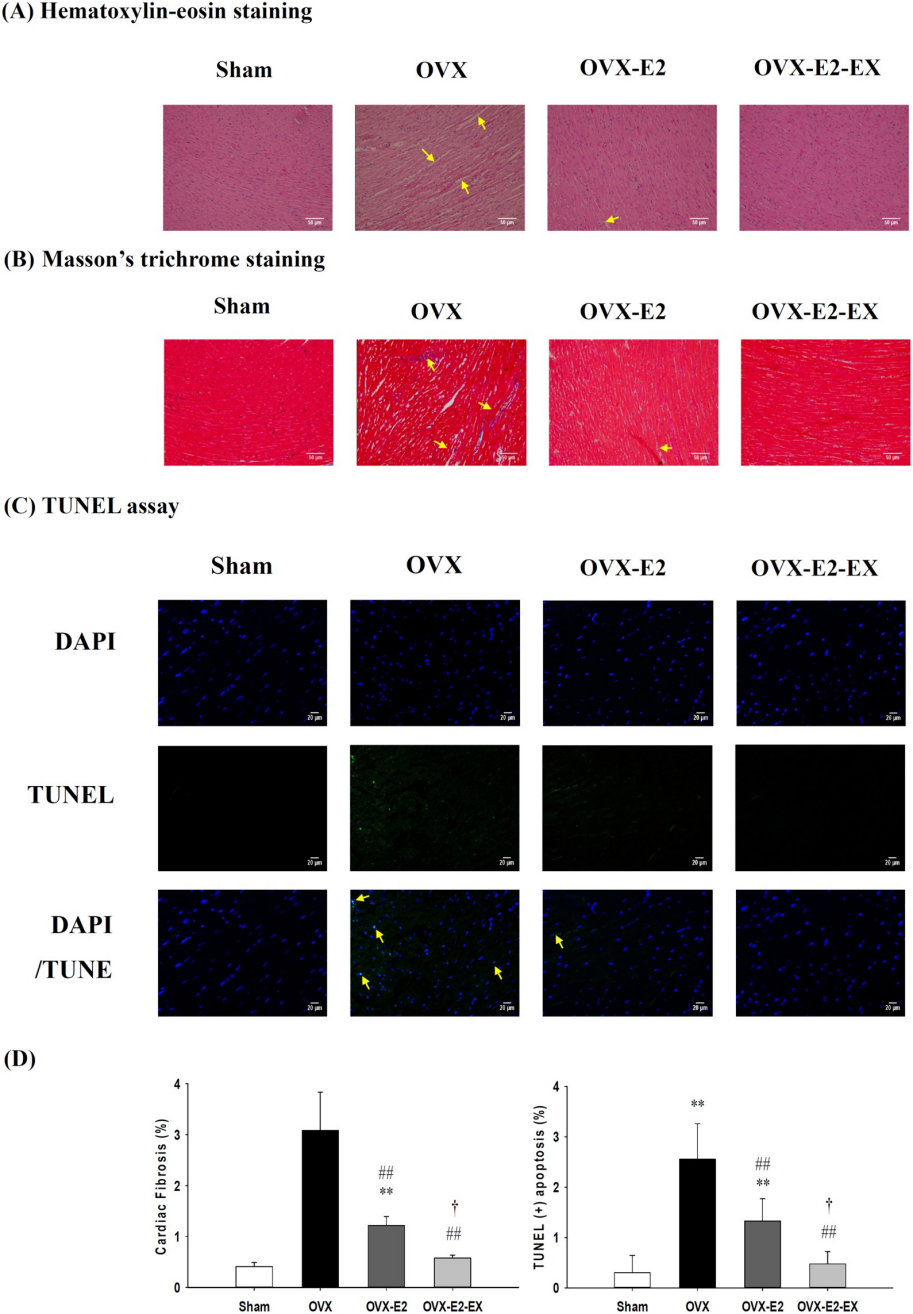
Effect of 17β-estradiol treatment and exercise training on cardiac histopathological and TUNEL-positive apoptotic changes. Representative histopathological analyses of cardiac sections from the left ventricles in the sham-operated (Sham), bilateral ovariectomized (OVX), OVX treated with 17β-estradiol (OVX-E2) and OVX rats treated with 17β-estradiol and exercise (OVX-E2-EX) groups were performed with (**A**) hematoxylin-eosin staining (interstitial space: wide, arrows indicated) and (**B**) Masson’s trichrome staining (fibrosis: blue color, arrows indicated). The images of the myocardial architecture are magnified 200×; (**C**) Representative stained apoptotic cells of cardiac sections were performed by staining with 4,6-diamidino-2-phenylindole (DAPI) staining (top, blue spots) and the terminal deoxynucleotidyl transferase dUTP-mediated nick-end labeling (TUNEL) assay (bottom, green spots, arrows indicated) The images of myocardial architecture are magnified 400×; (**D**) The bar represents the percentage of the blue area to the field area in Masson’s trichrome staining, and the percentage of TUNEL apoptosis was expressed as the ratio of TUNEL-positive cells relative to total DAPI-stained nuclei and indicates mean values ± SD (*n* = 6 in each group); **P<0.01, denotes significant differences from the Sham group. ^##^*P*<0.01, denotes the significant differences from the OVX group. ^†^*P*<0.05, indicates significant differences between the OVX-E2 and OVX-E2-EX group.

To investigate the co-effects of the 17β-estradiol (E2) treatment and exercise training on cardiac architecture, fibrosis and apoptosis after an ovariectomy, we conducted a histopathological analysis of the heart slices with Hematoxylin-Eosin staining, Masson’s trichrome staining, TUNEL and DAPI staining in hearts from the Sham, OVX, and OVX-E2 and OVX-E2-EX groups. The left ventricles of the OVX group showed abnormal myocardial architecture with increased cardiac interstitial space, fibrosis and increased TUNEL-positive apoptotic cells relative to the Sham group, whereas those showed a significant decrease in the OVX-E2 or OVX-E2-EX groups when compared with the OVX group, and even greater decreases were observed in the OVX-E2-EX group when compared with the OVX-E2 group (Fig 1A, 1B, 1C and 1D).

### Cardiac estrogen receptor and IGF-1 related pro-survival pathway

**Fig 2:**
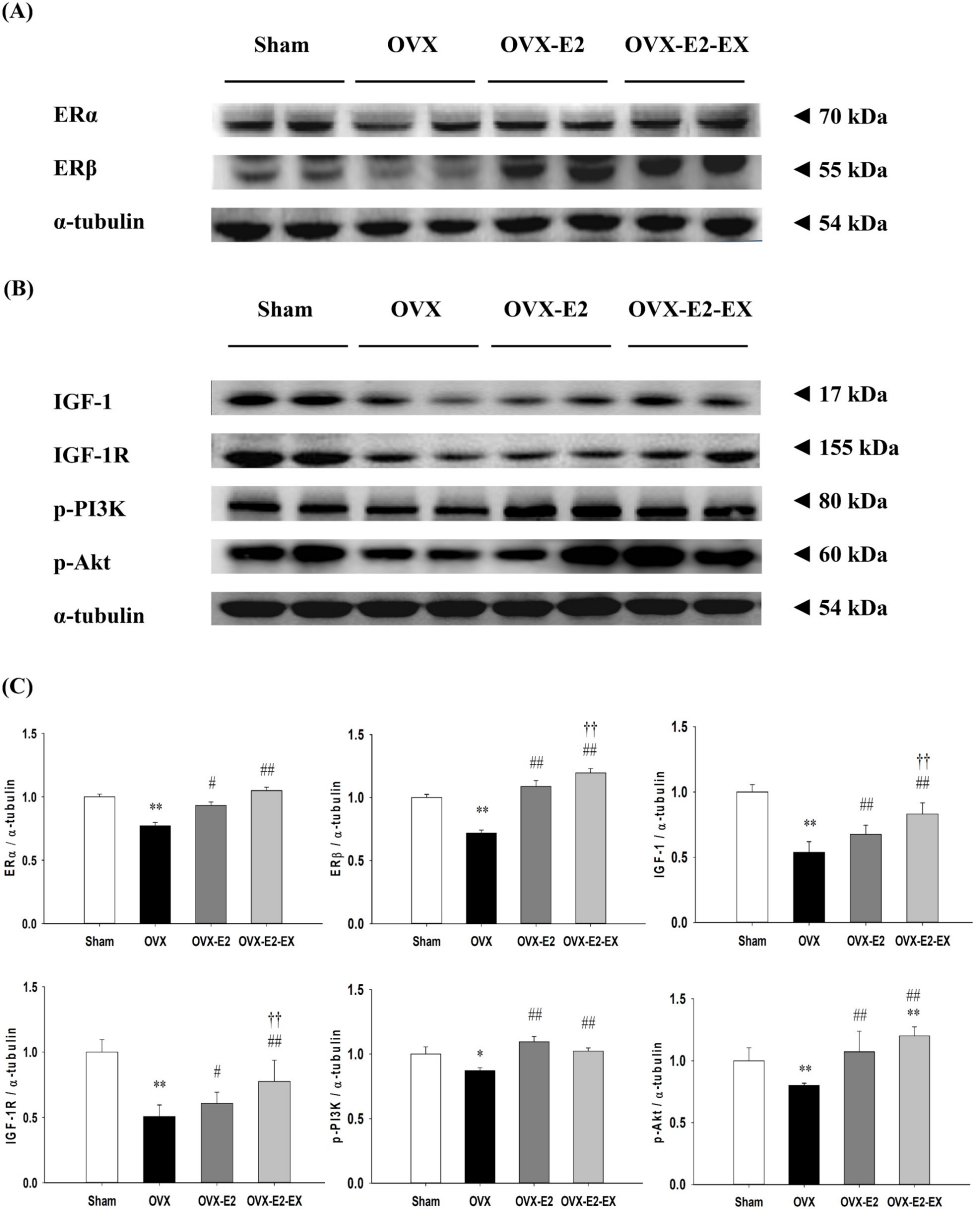
Effects of the 17β-estradiol treatment and exercise training on the cardiac estrogen receptor and IGF-1-related pro-survival pathways. **(A)** The representative protein products of estrogen receptor-α (ERα), estrogen receptor-β (ERβ), IGF-1, IGF-1R, p-PI3K and p-Akt extracted from the left ventricles of excised hearts of sham-operated (Sham), bilateral ovariectomized (OVX), OVX treated with 17β-estradiol (OVX-E2) and OVX rats treated with 17β-estradiol and exercise (OVX-E2-EX) were measured by Western Blotting analysis. **(B)** The bars represent the relative protein quantification of ERα, ERβ, IGF-1, IGF-1R, p-PI3K and p-Akt on the basis of α-tubulin and indicate the mean values ± SD (n = 8 in each group). * *P<*0.05, ** *P<*0.01 indicates significant differences from the Sham group. ^#^ *P<*0.05 and ^##^ *P<*0.01 indicates significant differences from the OVX group. ^††^ *P*<0.01, indicates significant differences between OVX-E2 and OVX-E2-EX group.

To investigate the co-effects of the E2 treatment and exercise training on the cardiac estrogen receptor and IGF-1-related pro-survival pathways after an ovariectomy, the protein levels of ERα, ERβ, IGF-1, IGF-1R, p-PI3K and p-Akt in the left ventricles excised from the Sham, OVX, and OVX-E2, and OVX-E2-EX groups were measured by Western Blotting. The estrogen receptor and pro-survival protein level of ERα, ERβ, IGF-1, p-PI3K and p-Akt were significantly decreased in the OVX group when compared with those in the Sham group, whereas those were significantly increased in the OVX-E2 or OVX-E2-EX groups (Fig 2). The protein levels of ERβ, IGF-1 and IGF-1R were even greater increases observed in the OVX-E2-EX group when compared with the OVX-E2 group (Fig 2).

### Cardiac Bcl-2 family survival pathway

**Fig 3:**
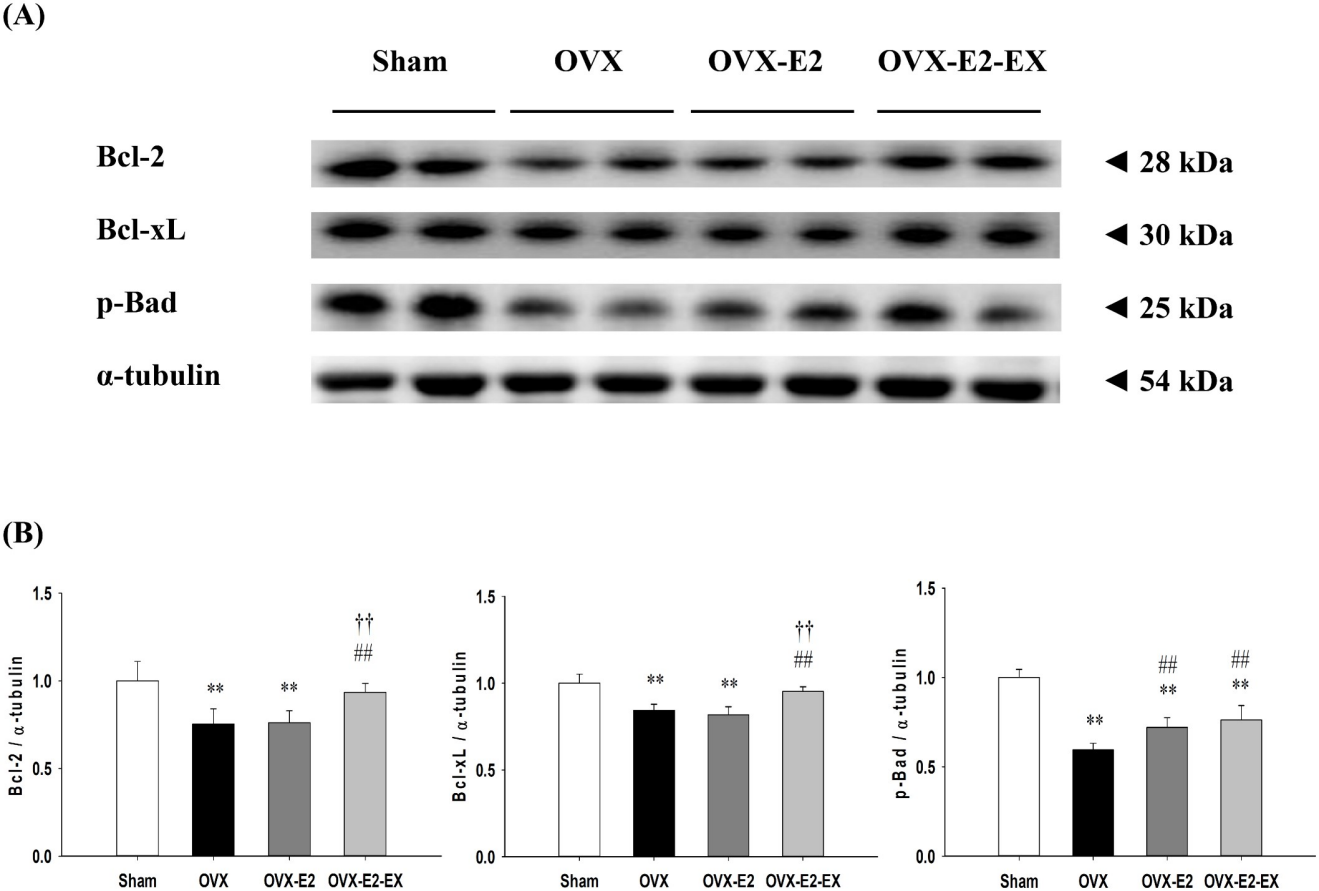
Effects of the 17β-estradiol treatment and exercise training on the cardiac anti-apoptotic pathway. **(A)** The representative protein products of Bcl2, Bcl-xL and p-Bad were extracted from the left ventricles of excised hearts of sham-operated (Sham), bilateral ovariectomized (OVX), OVX treated with 17β-estradiol (OVX-E2) and OVX rats treated with 17β-estradiol and exercise (OVX-E2-EX) were measured by Western Blotting analysis. **(B)** The bars represent the relative protein quantification of Bcl2, Bcl-xL and p-Bad on the basis of α-tubulin and indicate the mean values ± SD (n = 8 in each group). ** *P<*0.01 indicate significant differences from the Sham group. ^##^ *P<*0.01 indicate significant differences from the OVX group. ^††^ *P*<0.01, indicate significant differences between the OVX-E2 and OVX-E2-EX group.

To investigate the co-effects of the E2 treatment and exercise training on the cardiac anti-apoptotic pathways after an ovariectomy, the protein levels of Bcl-2, Bcl-xL and p-Bad in the left ventricles excised from the Sham, OVX, and OVX-E2, and OVX-E2-EX groups were measured by Western Blotting. The protein levels of Bcl-2, Bcl-xL and p-Bad were significantly decreased in the OVX group relative to the Sham group, whereas only the p-Bad was significantly increased in the OVX-E2 group when compared with the OVX group (Fig 3). The protein levels of Bcl-2, Bcl-xL and p-Bad were significantly increased in the OVX-E2-EX group when compared with the OVX group (Fig 3). Furthermore, when compared with the OVX-E2 group, the protein levels of Bcl-2 and Bcl-xL were even further increased in the OVX-E2-EX group (Fig 3).

### Cardiac mitochondria-dependent apoptotic pathways

**Fig 4:**
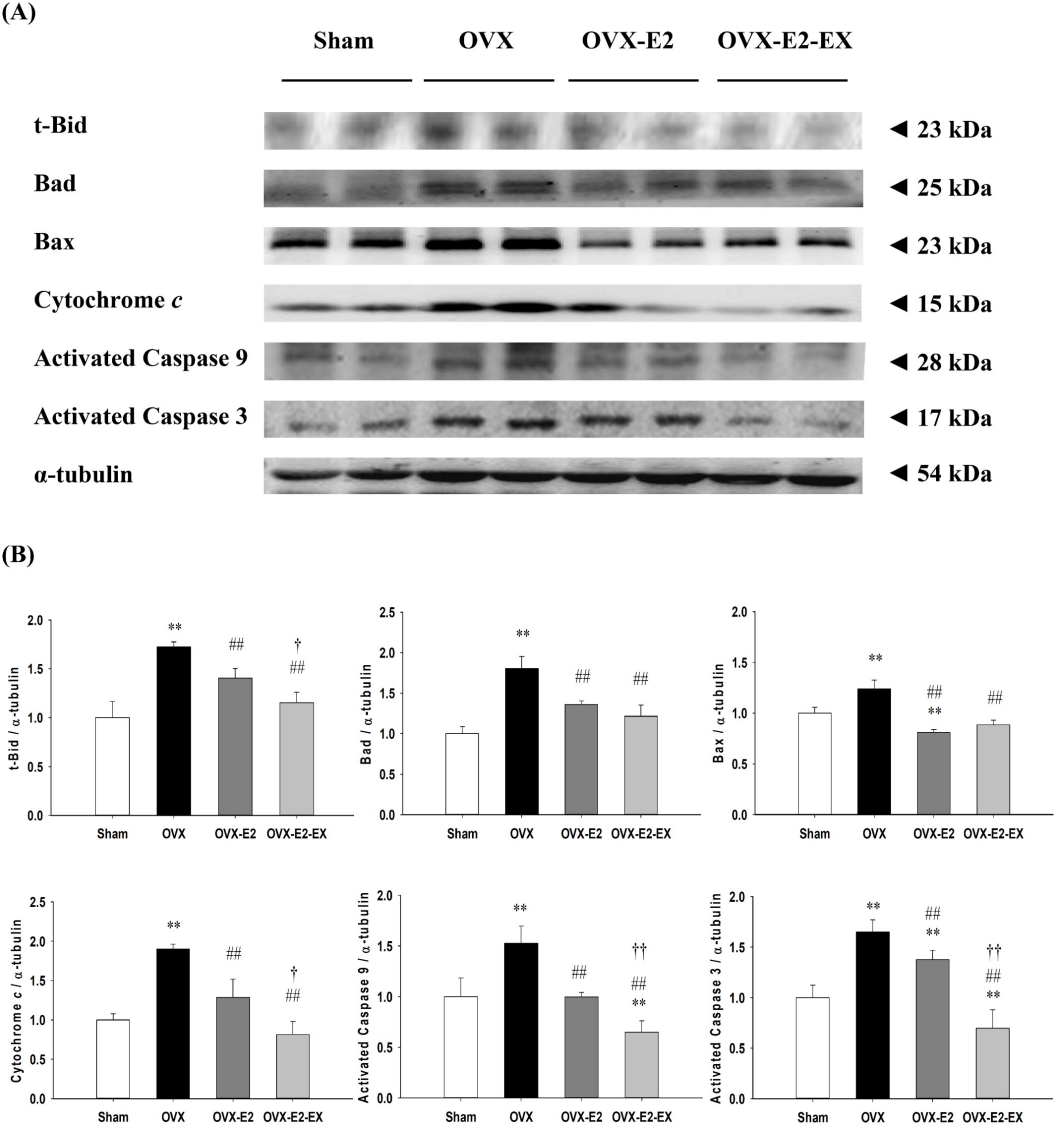
Effect of 17β-estradiol treatment and exercise training on cardiac mitochondria–dependent apoptotic pathway. **(A)** The representative protein products of t-Bid, Bad, Bax, Cytochrome *c*, activated caspase 9 and activated caspase 3 were extracted from the left ventricles of excised hearts of the sham-operated (Sham), bilateral ovariectomized (OVX), OVX treated with 17β-estradiol (OVX-E2) and OVX rats treated with 17β-estradiol and exercise (OVX-E2-EX) were measured by Western Blotting analysis. **(B)** The bars represent the relative protein quantification of t-Bid, Bad, Bax, and Cytochrome *c*, activated caspase 9 and activated caspase 3 on the basis of α-tubulin and indicate mean values ± SD (n = 8 in each group). **P<0.01, indicate significant differences from the Sham group. ^##^*P*<0.01, indicate significant differences from the OVX group. ^†^*P*<0.05, ^††^ *P*<0.01, indicate significant differences between the OVX-E2 and OVX-E2-EX group.

To further understand the co-effects of the E2 treatment and exercise training on the cardiac mitochondria-dependent apoptotic signaling pathways after an ovariectomy, the protein levels of t-Bid, Bad, Bax and Cytochrome *c* in the left ventricles excised from the Sham, OVX, and OVX-E2, and OVX-E2-EX groups were measured by Western Blotting. When compared with the Sham group, the protein levels of t-Bid, Bad, Bax, Cytochrome *c*, activated caspase 9 and activated caspase 3 were significantly increased in the OVX, whereas those were significantly decreased in the OVX-E2 or OVX-E2-EX groups when compared with the OVX group (Fig 4). The protein levels of t-Bid, Cytochrome *c*, activated caspase 9 and activated caspase 3were even further reduced in the OVX-E2-EX group when compared with the OVX-E2 group (Fig 4).

### Cardiac Fas receptor-dependent apoptotic pathway

**Fig 5:**
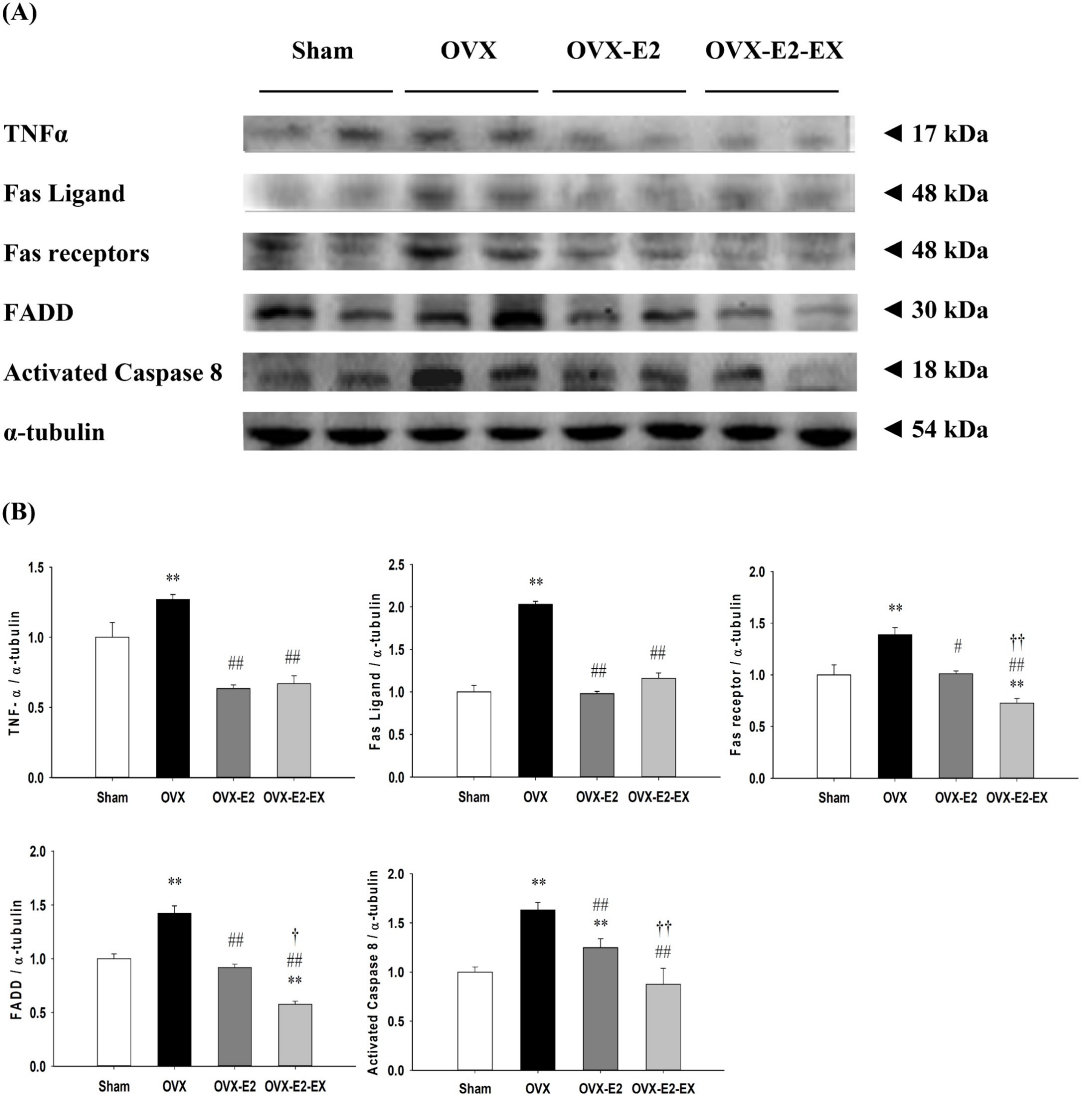
Effect of 17β-estradiol treatment and exercise training on cardiac Fas receptor–dependent apoptotic pathway. (A) The representative protein products of TNF-α, Fas ligand, Fas receptors, Fas-associated death domain (FADD) and activated caspase 8 were extracted from the left ventricles of excised hearts of the sham-operated (Sham), bilateral ovariectomized (OVX), OVX treated with 17β-estradiol (OVX-E2) and OVX rats treated with 17β-estradiol and exercise (OVX-E2-EX) were measured by Western Blotting analysis. **(B)** The bars represent the relative protein quantification of TNF-α, Fas ligand, Fas receptors, FADD and activated caspase 8 on the basis of α-tubulin and indicate the mean values ± SD (n = 8 in each group). **P<0.01, indicate significant differences from the Sham group. ^#^*P*<0.05, ^##^*P*<0.01, indicate significant differences from the OVX group. ^†^*P*<0.05, ^††^*P*<0.01, indicate significant differences between the OVX-E2 and OVX-E2-EX group.

To investigate the co-effects of the E2 treatment and exercise training on the cardiac Fas receptor-dependent apoptotic signaling pathways after an ovariectomy, the protein levels of the tumor necrosis factor-α (TNF-α), Fas ligand, Fas receptors, Fas-associated death domain (FADD) and activated caspase 8 in the left ventricles excised from the Sham, OVX, and OVX-E2, and OVX-E2-EX groups were measured by Western Blotting. When compared with the Sham group, the protein levels of TNF-α, Fas ligand, Fas receptors, FADD and activated caspase 8 were significantly increased in the OVX group, whereas those were significantly decreased in the OVX-E2 or OVX-E2-EX groups when compared with the OVX group (Fig 5). The protein levels of Fas receptors, FADD and activated caspase 8 were even further reduced in the OVX-E2-EX group when compared with the OVX-E2 group (Fig 5).

## Discussions

**Fig 6:**
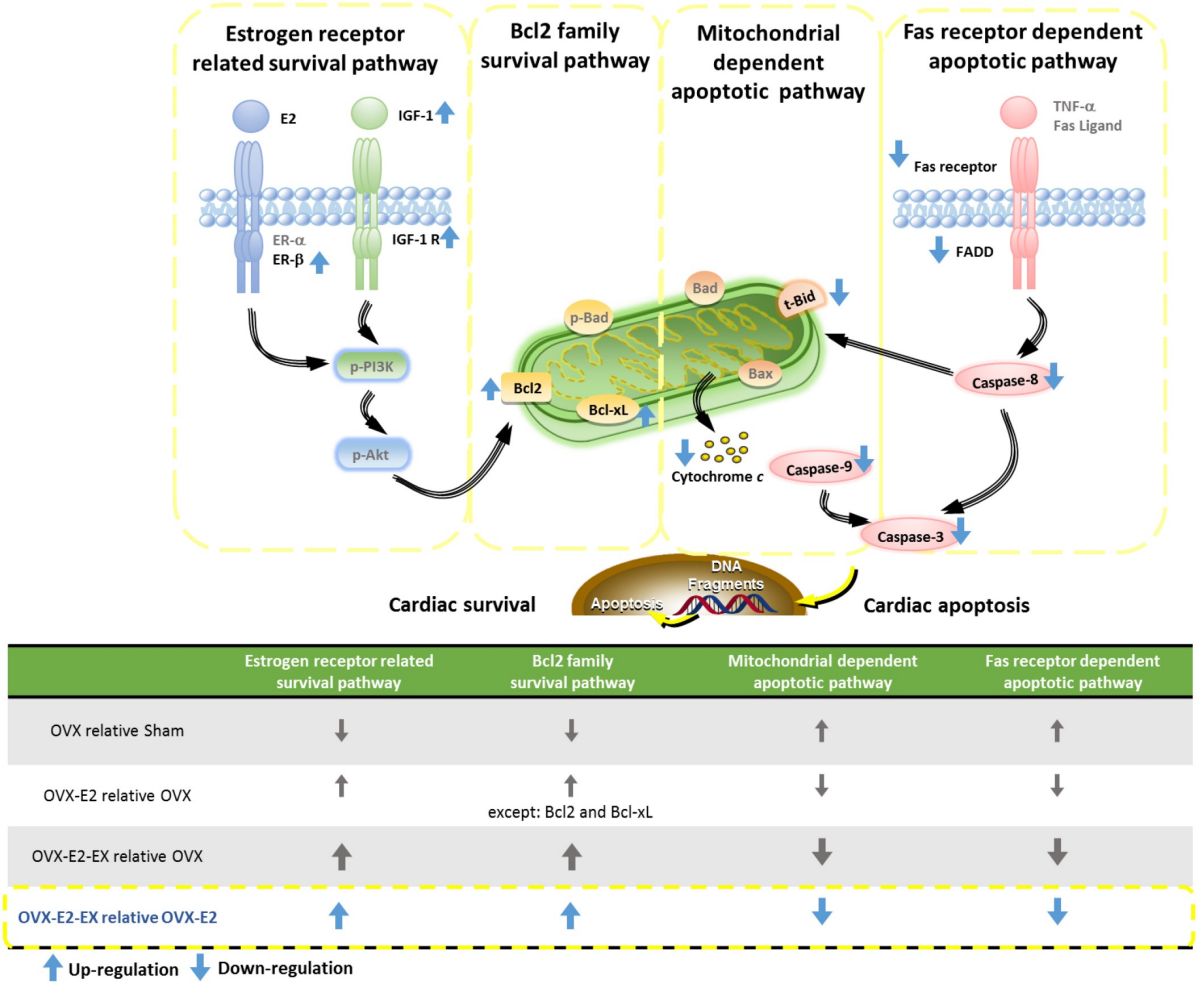
Proposed hypothesized diagram. Our hypothesis proposed that ovariectomy attenuated the estrogen receptor related survival pathway (ERα, ERβ, IGF-1, IGF-1R, p-PI3K, p-Akt, Bcl-2, Bcl-xL and p-Bad) and activated the cardiac mitochondria-dependent apoptotic pathway (t-Bid, Bad, Bax, Cytochrome *c*, activated caspase-9, and activated caspase-3), as well as activated the cardiac Fas receptor-dependent apoptotic pathway (TNF-α, Fas ligand, Fas receptors, activated caspase-8, and activated caspase-3) after an ovariectomy. However, the estrogen receptor enhanced the related survival pathway and suppressed the cardiac Fas receptor-dependent as well as the mitochondria-dependent apoptotic pathway which were observed in either the 17β-estradiol (E2) treatment or E2 treatment combined with exercise intervention. Furthermore, the protein levels of ERβ, IGF-1, IGF-1R, Bcl-2 and Bcl-xL were further enhanced when combined with E2 and exercise training. The protein levels of t-Bid, Cytochrome *c*, Fas receptors, FADD, activated caspase-8, activated caspase-9 and activated caspase-3 were further decreased when combined with E2 and exercise training.

Our main findings can be summarized as follows: 1) Combined 17β-estradiol and exercise training show a positive effect on the prevention from abnormal myocardial architecture enlarged interstitial space, increased cardiac fibrosis, and more cardiac TUNEL-positive apoptotic cells after an ovariectomy. 2) The cardiac estrogen receptor and IGF-1 and Bcl-2 family survival pathways after an ovariectomy were activated in either an 17β-estradiol treatment or 17β-estradiol treatment combined with exercise intervention, which is based on an increase in ERα, ERβ, IGF-1, IGF-1R, p-PI3K, p-Akt and p-Bad. 3) The cardiac mitochondria-dependent apoptotic pathway after the ovariectomy were attenuated in either the 17β-estradiol treatment or 17β-estradiol treatment combined with exercise intervention, with evidence of a decrease in t-Bid, Bad, Bax, Cytochrome *c*, activated caspase-9, and activated caspase-3. 4) The cardiac Fas receptor-dependent apoptotic pathways after an ovariectomy were attenuated in either the 17β-estradiol treatment or 17β-estradiol treatment combined with exercise intervention, with evidence of a decrease in TNF-α, Fas ligand, Fas receptors, FADD, activated caspase-8 and activated caspase-3. 5) Furthermore, the combined intervention of the 17β-estradiol treatment and exercise show positive effects, with evidence of enhancement in ERβ, IGF-1, IGF-1R, Bcl-2 and Bcl-xL, as well as attenuates in t-Bid, Cytochrome *c*, Fas receptors, FADD, activated caspase 8, activated caspase-9 and activated caspase-3 when compared with the treating of the 17β-estradiol treatment alone. Our results suggested that exercise training may enhance the effects of 17β-estradiol on pro-survival and anti-apoptosis in ovariectomized rats (Fig 6). Our findings support our hypothesis that the combined effect of the 17β-estradiol treatment and exercise training might be more effective than the 17β-estradiol treatment only in preventing mitochondria-dependent and Fas receptor-dependent apoptotic pathways through enhancing the ERβ-related survival pathways in ovariectomized rats.

Menopause-related estrogen-deficiency may cause cardiovascular remodeling and dysfunction, characterized by increased left ventricular hypertrophy, left ventricular dilatation and collagen deposition [2, 15, 26]. Those pathological characteristics were recognized as the most important predictor of cardiovascular morbidity and mortality and were important risk factors for heart failure [27]. In the present study, we observed that the ovariectomized rats suffered from increased left ventricular weight, abnormal myocardial architecture, enlarged interstitial space and more cardiac fibrosis.

17β-estradiol is known to provide a protective effect on the cardiovascular system [13]. A study reported that about a 50% reduction in cardiovascular events in women receiving postmenopausal hormone replacement therapy, when compared with untreated women [28]. A previous study has indicated that women receiving hormone replacement therapy presented a reduction of the left ventricular mass and left ventricular dimensions when compared with women without hormone replacement therapy [29]. Moreover, hormone replacement therapy has been shown to attenuate cardiac remodeling and reducing interstitial myocardial fibrosis after an ovariectomy or aging-related defects [14, 15]. However, previous study indicated that physiological levels of 17β-estradiol (E2, 6μg/kg or 20μg/kg) exhibit neuroprotective effects on cerebral ischemia; whereas, supraphysiological doses of 17β-estradiol (E2, 50μg/kg) have damaging effects on neurons after cerebral ischemia [30]. 17β-estradiol concentrations cause neurologic damaging effects when administered dose leads to a blood 17β-estradiol level exceed normal physiologic level (physiologic range >50pg/mL) [31]. In current study, 17β-estradiol was administered after ovariectomy at a low doses of 10 μg/kg, which does not exceed the physiologic concentration. Nevertheless, long-term 17β-estradiol therapy is associated with an increased risk of breast cancer, or severe side effects in some women [16, 17]. Several research suggested that alternative therapeutic approaches need be discovered for menopausal women. Exercise training has beneficial effects on the cardiovascular system for menopausal women [20, 22, 32]. Our recent study also showed that exercise training prevents cardiac fibrosis and cardiac apoptosis in ovariectomized rats [23]. The current study further showed that the 17β-estradiol treatment and moderate exercise training appeared to have positive effects on the improvement of cardiac myocardial architecture through prevention of apoptosis and fibrosis. Previous study reported that aerobic exercise can significantly change estrogen metabolism in premenopausal women, which may lower breast cancer risk. The study suggested that exercise intervention can lead to increases in 2-hydroxyestrone (2-OHE1) and possible decreases in 16α-hydroxyestrone (16α-OHE1) ultimately resulting in significant increases the ratio of the estradiol metabolites 2-OHE1/16α-OHE1 in premenopausal women, which may lower breast cancer risk [24]. It implies possibly that 17β-estradiol treatment plus exercise training not only diminished cardiac apoptosis and fibrosis, but also decreased risk for estrogen related disease ex. breast cancer, which might provide one of new therapeutic effect on cardiac protection in menopausal women treated with 17β-estradiol, even though we haven’t measure estrogen metabolism in current study.

It has been well known estrogen receptor alpha (ERα) and beta (ERβ) are involved in the regulation of pathological myocardial hypertrophy. Our previous study suggested that ovariectomy-induced cardiac widely dispersed apoptosis might be potentially associated with chronic heart failure or cardiovascular dysfunction in menopausal women [8]. Another study reported that ovariectomy–induced cardiac apoptosis and heart remodeling through attenuated estrogen receptor ERα and ERβ [15, 25]. Widely dispersed apoptosis occurring in terminally differentiated cardiomyocytes is a crucial pathological mechanism leading to chronic heart failure [4, 5]. The present study focuses on understanding the process of anti-apoptotic and pro-survival pathways which could allow for the development of more effective therapeutic novel strategies to reverse or attenuate heart failure through combined hormonal therapy and exercise therapy. 17β-estradiol treatment may enhance the PI3K/Akt signaling dependent pro-survival pathways and suppressed cardiomyocyte apoptosis in the observed estrogen-mediated reduction in myocardial injury [12]. The IGF-1/PI3K signaling is a crucial mediator in exercise training-induced physiological heart growth [33, 34]. Previous study indicated that exercise training can possibly reverse cardiac molecular and functional abnormalities which are associated with enhanced IGFI-R/PI3K/Akt and Bcl-2 family associated pro-survival pathways [35]. Exercise-induced physiological myocardial hypertrophy in females is mediated by induction of AKT signalling, MAPK-pathways, protein synthesis and mitochondrial adaptation via ERβ [36]. In current studies, we observed that 17β-estradiol combined with exercise training exhibits positive effect of protection on ovariectomy-induced cardiac mitochondria-dependent and Fas receptor-dependent apoptosis. It implies possibly that the co-effects of exercise training and 17β-estradiol would be more effective than 17β-estradiol treatment alone to prevent ovariectomy-induced widely dispersed apoptosis. Furthermore, we further observed that the 17β-estradiol treatment when combined with exercise training, appeared to sub-additively enhance cardiac ER-β associated pro-survival pathways, indicating that ERβ showed an effect of 17β-estradiol treatment and exercise training beyond that noted for ERα, that showed mainly an effect of exercise training. It implies possibly that exercise training enhances the physiological effect of the 17β-estradiol treatment through up-regulation of the ER-β-mediated IGF-1 and Bcl-2 family survival pathways could be more effective to prevent cardiac apoptosis. Our findings from an ovariectomized animal model further hypothesize that regular exercise in postmenopausal women who receive estrogen may more effective to interrupt cardiac apoptosis and prevent the development of heart failure. Our results suggest that menopausal women who used the 17β-estradiol treatment recommendation combined with aerobic exercise training were hypothesized to derive greater benefit on cardiac function than menopausal women who used the 17β-estradiol treatment alone. In addition, by using a similar speed to previous study, a speed was 24 m/min, which corresponds to about 50–60% VO_2max_ [37]. Several studies have suggested that the regular practice of moderate-intensity exercise training has been shown many favorable cardiovascular benefits in menopausal rat or human model [20, 23]. This implies that all subjects could be engaged in moderate-intensity exercise training. In sum, menopausal women should be highly aware of the progressive development in cardiac abnormality or heart failure, as well as they should devote themselves to exercise training and lifestyle modification.

Most women became overweight or obese during the menopause transition stage and consequently, increase the potential of many cardiovascular risks [38]. Weight gain or Obesity may also lead to cardiac widely dispersed apoptosis. However it is still unclear whether the increased cardiac apoptotic activity is partially due to the deleterious factor of weight gain after ovariectomy [39–41]. Nevertheless, in our current study, we show that body weight can be partially decreased when combined with 17β-estradiol and exercise training, and even diminished cardiac apoptotic activity after an ovariectomy. We might further hypothesize that the combined intervention of 17β-estradiol treatment and exercise is a therapeutic agent for preventing overweight or reducing obesity-related cardiovascular health problems in menopausal obese women.

There are some limitations in this study. This study was to better understand whether the combined effect of the 17β-estradiol treatment and exercise training might be more effective than the 17β-estradiol treatment alone in preventing cardiac widely dispersed apoptosis. Therefore, this study did not include an exercise group alone. Because some postmenopausal women still need taking 17β-estradiol to ease menopausal symptoms, but long-term 17β-estradiol therapy alone is associated with increased risk for estrogen related disease ex. breast cancer, or severe side effects in some women [16, 17]. This study provide another one possible therapeutic advantage and development of more effective therapeutic novel strategies to reverse or attenuate heart failure through combined hormonal therapy and exercise therapy. This implies that further therapeutic treatment could be reduced 17β-estradiol dose and combined exercise therapy, which might help prevent adverse side effects and further studies are required to evaluate this possible outcomes. Additionally, we indicating that inhibit the cardiac apoptotic cascade and enhance survival signals are associated with ERβ, which showed mainly an effect of exercise training. A previous study has indicated exercise-induced physiological effect in females is mediated by induction of AKT signalling, MAPK-pathways, protein synthesis and mitochondrial adaptation via ERβ [36]. This implies possibly that MAPK-pathway may be associated with inhibit the cardiac apoptotic cascade and enhance survival signals. However, the mechanism of MAPK-pathway in ovariectomy was not determined in this work. Future studies will be carried out to investigate the importance and existence of these potential mechanisms.

### Hypothesized and clinical application

Cardiac tissues are difficult to be extracted from humans’ hearts, the current bilateral oophorectomy in the animal model should provide an important mechanism for explaining cardiac diseases in women with postmenopausal or early oophorectomy. In conclusion, our current findings revealed that the 17β-estradiol treatment combined with exercise training was more effective than 17β-estradiol treatment alone to prevent ovariectomy-induced widely dispersed apoptosis by enhancing ERβ-related survival pathways. Exercise training enhances the effect of the 17β-estradiol treatment which might provide one possible mechanism to anti-apoptotic strategies and prevent the development of heart failure in postmenopausal women. Hence, we might further hypothesize that the 17β-estradiol treatment combined with exercise training may have a therapeutic advantage over monotherapy, which may be one of several important therapeutic approaches to prevent cardiomyocyte apoptosis when menopausal women treated with 17β-estradiol clinically. Of course, further therapeutic or clinical studies are required to clarify any possible therapeutic application.
